# Progressive Degradation of Crude Oil *n*-Alkanes Coupled to Methane Production under Mesophilic and Thermophilic Conditions

**DOI:** 10.1371/journal.pone.0113253

**Published:** 2014-11-19

**Authors:** Lei Cheng, Shengbao Shi, Qiang Li, Jianfa Chen, Hui Zhang, Yahai Lu

**Affiliations:** 1 College of Resources and Environmental Sciences, China Agricultural University, Beijing, 100193, China; 2 Key Laboratory of Development and Application of Rural Renewable Energy, Biogas Institute of Ministry of Agriculture, Chengdu, 610041, China; 3 State Key Laboratory of Petroleum Resources and Prospecting, China University of Petroleum (Beijing), Beijing, 102200, China; University of Kansas, United States of America

## Abstract

Although methanogenic degradation of hydrocarbons has become a well-known process, little is known about which crude oil tend to be degraded at different temperatures and how the microbial community is responded. In this study, we assessed the methanogenic crude oil degradation capacity of oily sludge microbes enriched from the Shengli oilfield under mesophilic and thermophilic conditions. The microbial communities were investigated by terminal restriction fragment length polymorphism (T-RFLP) analysis of 16S rRNA genes combined with cloning and sequencing. Enrichment incubation demonstrated the microbial oxidation of crude oil coupled to methane production at 35 and 55°C, which generated 3.7±0.3 and 2.8±0.3 mmol of methane per gram oil, respectively. Gas chromatography-mass spectrometry (GC-MS) analysis revealed that crude oil *n*-alkanes were obviously degraded, and high molecular weight *n*-alkanes were preferentially removed over relatively shorter-chain *n*-alkanes. Phylogenetic analysis revealed the concurrence of acetoclastic *Methanosaeta* and hydrogenotrophic methanogens but different methanogenic community structures under the two temperature conditions. Candidate divisions of JS1 and WWE 1, *Proteobacteria* (mainly consisting of *Syntrophaceae*, *Desulfobacteraceae* and *Syntrophorhabdus*) and *Firmicutes* (mainly consisting of *Desulfotomaculum*) were supposed to be involved with *n*-alkane degradation in the mesophilic conditions. By contrast, the different bacterial phylotypes affiliated with *Caldisericales*, “Shengli Cluster” and *Synergistetes* dominated the thermophilic consortium, which was most likely to be associated with thermophilic crude oil degradation. This study revealed that the oily sludge in Shengli oilfield harbors diverse uncultured microbes with great potential in methanogenic crude oil degradation over a wide temperature range, which extend our previous understanding of methanogenic degradation of crude oil alkanes.

## Introduction

Crude oil is a complex mixture containing many thousands of different hydrocarbon compounds, which can be divided into four classes (saturated hydrocarbons, aromatic hydrocarbons, asphaltene and non-hydrocarbons). The biodegradation of crude oil by natural populations of microorganisms was reported over a century ago. Under aerobic conditions, general progressive biodegradation of petroleum hydrocarbon proceeds first with loss of *n*-alkanes, then isoprenoids, cyclic alkanes, and lower molecular weight aromatics, followed by the remainder of the more complex, higher molecular weight constituents [1], [2]. Over the past two decades, it has consistently been shown that crude oil hydrocarbons could be degraded under nitrate-reducing [3], ferric iron-reducing [4], sulfate-reducing [5], [6] and methanogenic conditions [7]–[9]. Multiple research teams have reported that the entire *n*-alkane fraction of crude oil can be consumed under sulfate reducing and/or methanogenic conditions [6], [9]–[13]. Rueter *et al*. [5] reported for the first time that C_8_ to C_11_ *n*-alkanes of crude oil were completely degraded, and C_12_ to C_16_ *n*-alkanes were partially consumed by a thermophilic sulfate-reducing bacterium, but degradation of alkanes above hexadecane was not observed. Rabus *et al*. [14] enriched a denitrifying culture capable of degrading C_1_–C_3_ alkylbenzenes in the first generation incubation and growing on C_5_–C_20_ *n*-alkanes and alkylbenzenes in the second subculture. Siddique *et al*. [15],[16] reported the preferential use of *n*-alkanes *n*C_10_> *n*C_8_> *n*C_7_> *n*C_6_, but no preferential degradation of longer-chain *n*-alkanes (C_14_, C_16_ and C_18_) occurred. Additionally, the initial loss of the longer-chain *n*-alkanes of crude oil under sulphate reducing and methanogenic conditions has also been reported [17]–[19].

The anaerobic degradation of non-methane hydrocarbons is different from the aerobic process. Anaerobic hydrocarbon degradation is activated by fumarate addition, carboxylation, methylation, hydroxylation [20]–[22] and potentially other unknown mechanisms [23]. Over twenty culturable bacteria have been isolated and characterized that are capable of alkane degradation with nitrate and sulfate as electron acceptors (Widdel *et al.* 2010 and references therein). The methanogenic conversion of crude oil hydrocarbons requires syntrophic communities of acetogenic bacteria and methanogenic archaea from the thermodynamics point of view [24]. To date, no pure syntrophic hydrocarbon degraders have been isolated with the exception of a sulfate reducer capable of syntrophic hexadecane degradation in coculture with hydrogenotrophic methanogens [25]. The development of culture-independent approaches has revealed the vast majority of as-yet-uncultured syntrophic bacterial species present in methanogenic hydrocarbon degrading consortia [8]. For example, uncultured members of the *Syntrophaceae* family have been implicated in syntrophic alkane degradation under mesophilic conditions using qPCR and DNA-SIP [26],[27]. *Thermotogae*- and *Firmicutes*-related members were the dominant phylotypes in thermophilic, methanogenic alkane degrading cultures [11],[28],[29]. Acetoclastic and hydrogenotrophic methanogens were always observed in the methanogenic communities, but research on the relative contribution of these two methanogenic pathways to total methane production during the anaerobic degradation of *n*-alkanes is limited [13],[27].

Temperature influences petroleum biodegradation by affecting the chemical composition of the oil, the rate of hydrocarbon metabolism by microorganisms, and the composition of the microbial community [30]. Previous studies reported that microbial oxidation of *n*-alkanes coupled to methane production could occur in the temperature range 20–55°C [7],[11],[13],[15],[28],[31]. Little is known about the potential for crude oil degradation and methane production under different temperature conditions, and how indigenous microbes respond to the crude oil and temperature shift. In this study, oily sludge microbes originating from Shengli oilfield were incubated at 35 and 55°C. The microbial activity of crude oil degradation was evaluated by detecting methane production and crude oil degradation over time. The microbial community structures were characterized using terminal restriction fragment length polymorphism (T-RFLP) fingerprinting and sequencing of 16S rRNA gene fragments.

## Materials and Methods

### Ethics statement

No specific permits were required for the described field studies. No specific permissions were required for these locations/activities. The sampled locations are not privately owned or protected in any way, and the studies did not involve endangered or protected species.

### Medium and incubation

#### Sample and medium

Oily sludge was sampled from a disposal field of Shengli oilfield, China, where oily sludge from oil tanks and pipelines in block Gudao of Shengli oil field was treated. The sludge samples were collected in February 2009, and stored at 4°C before experiments commenced. The dehydrated crude oil sampled from Block L801 in Shengli oilfield, with an average density of 0.926 g.mL^−1^, was autoclaved at 121°C for 30 min, and repeated three times within a week. Fresh medium without sulfate and nitrate was prepared according to a previous report using the Hungate anaerobic technique [32]. Aliquots of medium were distributed into glass vials sealed with isobutyl rubber stoppers (Bellco, USA) and aluminum caps under a gas atmosphere of 80% N_2_ and 20% CO_2_, in which resazurin (1 mg.L^−1^) was added as a redox indicator. Vials were autoclaved at 121°C for 30 min. Solutions of sterile Na_2_S.9H_2_O (0.03%), NaHCO_3_ (0.25%), vitamin solution (2 ml.L^−1^), trace elements solution SL-7 (2 ml. L^−1^), vitamin B_12_ (2 ml.L^−1^) and vitamin B_1_ (2 ml.L^−1^) [32] were injected into fresh medium before inoculation and the pH was adjusted to 7.0–7.2.

#### Methanogenic enrichment incubation

Approximately 50 g of oily sludge was dispersed into 600 mL vials (Fuxin, China) amended with 300 mL of fresh medium and *ca.* 3.2 g of sterile crude oil. The vials were incubated statically in the dark at 35 and 55°C to obtain pre-enrichment cultures. Three sets of enrichments were carried out in 120 mL glass vials containing 50 mL of freshwater medium: (1) the experiment group: 7.5 mL of pre-enrichment culture and 1 g of sterile crude oil, (2) the abiotic control group: 7.5 mL of pre-enrichment cultures; (3) the crude oil-free control group: 1 g of sterile crude oil. Numerous parallel cultures were prepared in each set, and incubated without shaking at 35 and 55°C in the dark. Two to three vials in each set were sacrificed for DNA extraction and/or crude oil determination at different time points.

### Chemical Analyses

#### Methane determination

The gas sample (0.2 mL) was sampled by a gas-tight syringe with a pressure lock (Vici, USA), and injected into a gas chromatograph equipped with a thermal conductivity detector (Shimadzu GC 2010, Kyoto, Japan) for methane determination [33]. The gas pressure in the culture vials was determined with a barometer (Aukis, Shanghai, China), and the amount of methane production was calculated based on Avogadro's law after calibration with a gas mixture of N_2_ (29.96%), CH_4_ (39.99%), and CO_2_ (30.05%).

#### Crude Oil Analysis

Crude oil (30–50 mg) was loaded into a silica gel column with neutral aluminum oxide (100–200 mesh). The column was subsequently eluted with *n*-hexane, methylene chloride: *n*-hexane (2∶1) and chloroform: ethanol (98∶2), to collect saturated hydrocarbons, aromatic hydrocarbons and non-hydrocarbons successively. The residues remaining in the column after elution contained the asphaltene fraction. The saturated hydrocarbons and internal standard (*d*_50_-*n*-tetracosane) were analyzed by a gas chromatograph (Agilent 7890A, Santa Clara, CA) using an HP-5 MS fused silica capillary column (60 m×250 µm×25 µm film thickness). The carrier gas was Helium (99.999%) at a flow rate of 1 ml.min^−1^. The temperature program was run from 50°C (1 min isotherm) to 120°C at 20°C per min, and further increased to 310°C at 3°C per min with a hold at 310°C for 25 min. Mass spectral data were generated by a mass spectrometer (Agilent 5975i) at an energy of 70 eV in SCAN/SIM mode.

### Microbial analysis

#### DNA Extraction and PCR Amplification

The liquid cultures (2–4 mL) were centrifuged for 5 min at 14000 rpm at 4°C, and the pellets were maintained at −80°C. Genomic DNA was extracted using a bead-beating method [34]. DNA fragments were purified with a Wizard DNA clean-up system (Promega, Madison, WI) and checked using 1% agarose gel electrophoresis. PCR amplifications of the archaeal and bacterial 16S rRNA gene fragments for terminal restriction fragment length polymorphism (T-RFLP) and 16S rRNA clone library were accomplished using primers A109f/A934r [35],[36] and B27f/B907r [37], respectively. The PCR amplifications of archaeal and bacterial 16S rRNA genes were performed as previously described [38].

#### T-RFLP Analysis

PCR amplifications for T-RFLP analysis used the same mixtures and programs as described above, but the 5′ end of primers A934r and B27f were labeled with 6-carboxyfluorescein (FAM) [39]. The FAM-labeled PCR products were purified with TIANquick Midi Purification Kit (TIANGEN, Beijing, China), then digested at 65°C for archaeal DNA using *Taq* I and 37°C for bacterial DNA using *Msp* I (TakaRa, Otsu, Japan) based on the manufacturer's instructions. The digestion products were further purified using the ethanol precipitation method [38]. Dried DNA samples were resuspended in 10 µL ddH_2_O and a portion of each sample was mixed with deionized formamide containing 2% (v/v) internal standard ROX 30–1000 (Bioventure, Murfreesboro, TN). The mixtures were denatured at 95°C for 4 min and chilled on ice for 10 min. The DNA fragments were separated by capillary electrophoresis on a Genetic Analyzer 3130*xl* (Applied Biosystems, Foster City, CA). The relative terminal restriction fragment (T-RF) abundances of representative phylotypes were analyzed with GeneMapper 4.0 (ABI). T-RFs with a peak height of less than 100 fluorescence units were excluded from analyses. Relative T-RFs abundance were determined as relative signal intensities of T-RFs with peak height analysis integration, and the relative abundance of T-RFs less than 2% were grouped together.

The experimental T-RFs were identified by comparison with *in silico* digested T-RFs using the clone library sequences generated from the same methanogenic consortia, or discriminated by T-RFLP analysis of 16S rRNA genes isolated from corresponding single colonies as templates. The relationship between methane production and bacterial T-RFs was assessed by redundancy analysis (RDA) using Canoco for Windows Version 4.5 [40]. T-RFs were assigned as species and methane production was the environmental variable. Significance of the factors was tested using Monte Carlo permutations (1499 permutations).

#### Construction of 16S rRNA Gene Clone Libraries

The PCR products were gel-purified with TIANgen Universal DNA Purification Kit (TIANGEN, China). The purified fragments were cloned into pMD 19-T vector (Takara, Otsu, Japan) and inserted into *E. coli* Competent Cells JM109 (Takara, Japan) according to the manufacturer's instructions. Single colonies were prepared and sequenced using a Genetic Analyzer 3730*xl* (ABI) as previously described [28].

#### Phylogenetic Analysis

The 16S rRNA gene sequences were checked with the “Chimera check with Bellerophon” program of the Greengene database [41]. The checked sequences were grouped into operational taxonomic units (OTUs) with a 97% threshold [42]. All sequences were submitted into the RDP database and classified into different taxonomic levels using RDP naïve Bayesian rRNA Classifier with an 80% confidence threshold [43], and representative sequences from each OTU were used to search for the most similar type strains using the Seqmatch program [44]. The diversity coverage of the 16S rRNA gene clone library was calculated using Good's formula as C =  [1−(n_1_/N)] ×100, where n_1_ is the number of unique OTUs and N is the total number of clones in the library [45]. The rarefaction curve was further generated using PAST version 2.00 [46]. Phylogenetic trees of the archaeal and bacterial 16S rRNA genes were created using the neighbor-joining method of Mega 5.1 [47]. Bootstrap values were calculated after 1,000 replications. Sequences were deposited in GenBank database with accession numbers JF946838–JF947178 and JN860886–JN860927.

## Results

### Methane Production

**Figure 1:**
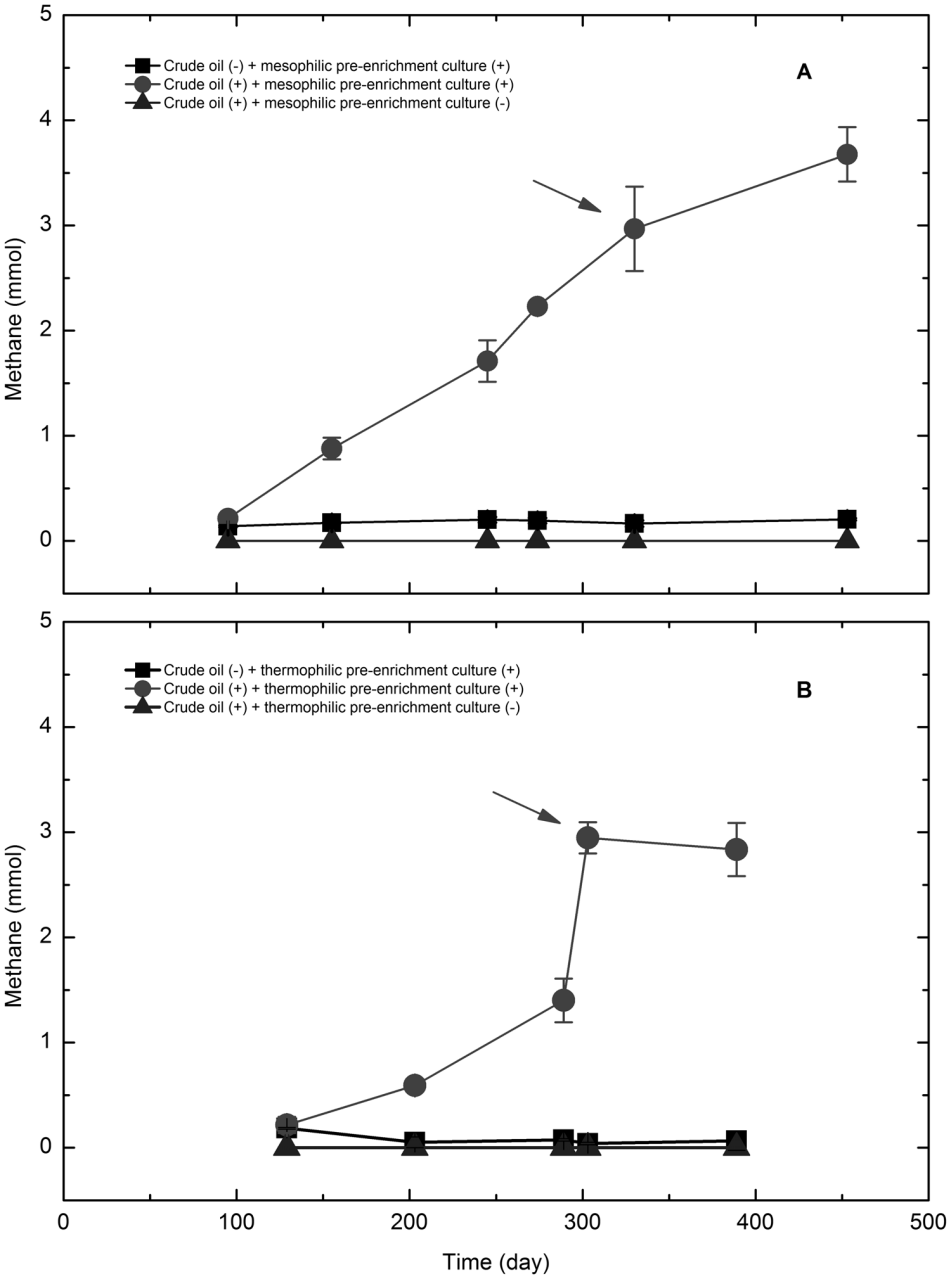
Time course of methane production at different temperatures. A: 35°C; B: 55°C. Arrows indicate sampling points for construction of clone libraries. • and ▴: Cultures were grown in triplicate, error bars represent the standard deviation. ▪: Cultures were grown in duplicate, error bars represent the standard deviation.

The pre-enrichment cultures accumulated methane consecutively at 35 and 55°C (Fig. S1 in File S1), indicating that the oily sludge microbes could grow at both temperatures. To further confirm methanogenic degradation of crude oil, the pre-enrichment cultures were subcultured and incubated at the same pre-enrichment temperature. The mesophilic culture (35°C), amended with crude oil, produced 3.7±0.3 mmol of methane with a maximum specific methane production rate of 0.01 mmol.d^−1^ after 453 days of incubation (Fig. 1A). The thermophilic consortium (55°C), amended with the same amount of crude oil, accumulated 2.8±0.3 mmol of methane after 389 days of incubation with maximum specific methane production rate of 0.02 mmol.d^−1^ (Fig. 1B). Less than 0.2 mmol methane was produced by the crude oil-free cultures at either temperature, and no methane was detected in the abiotic control.

### Biodegradation of Petroleum Hydrocarbon

**Figure 2:**
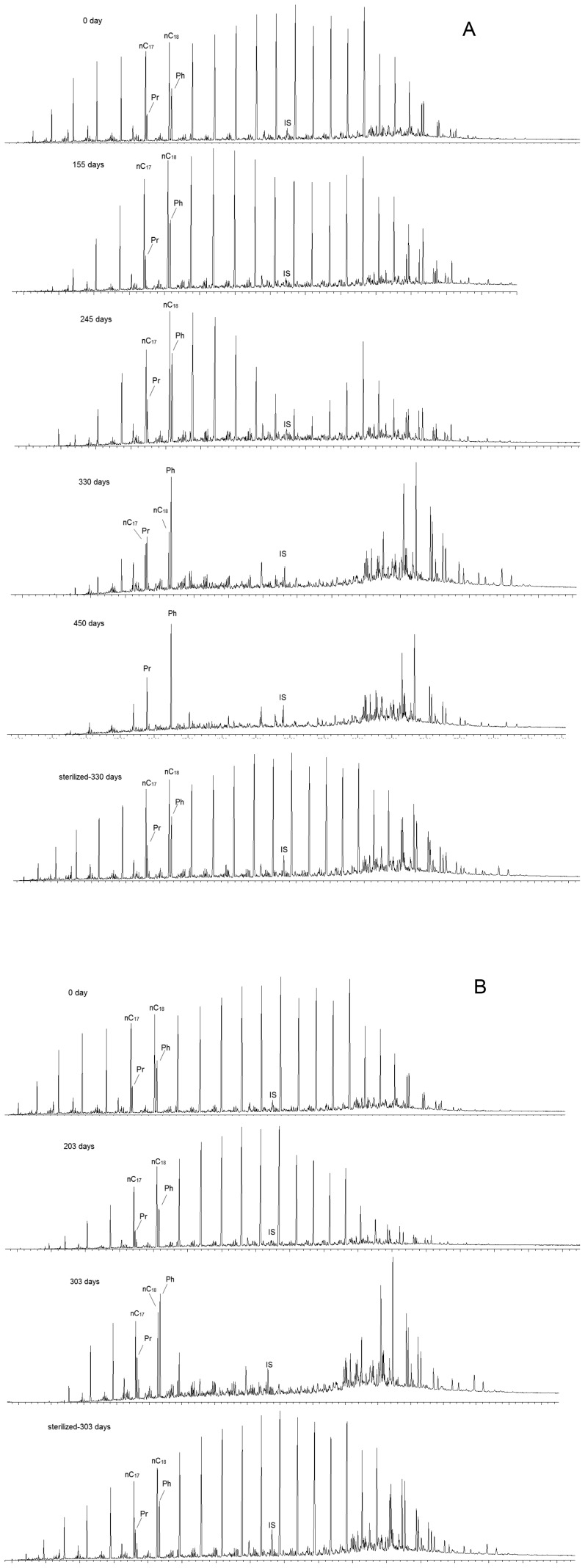
Gas chromatograms of the saturated hydrocarbon factions of crude oil. A: 35°C; B: 55°C. Pr: pristane; Ph: phytane; nC_17_: *n*-heptadecane; nC_18_: *n*-octadecane; IS: *d*_50_-*n*-tetracosane. The crude oil samples were collected from the crude oil-degrading cultures at different time points, with the exception that “sterilized-330 days” crude oil was sampled from the second control after 330 days of incubation at 35°C, and “sterilized-303 days” crude oil was sampled from the second control after 303 days of incubation at 55°C.

The continually accumulating methane generated in the crude oil-amended cultures relative to the oil-free controls indicated the biological degradation of crude oil via methanogenesis. The percentage of saturated hydrocarbons decreased from 33.4% to 8.6±0.9% at day 453 in the mesophilic consortium, and to 10.8±0.7% after 303 days of incubation in the thermophilic consortium (Table 1). From analysis of the saturated hydrocarbon profiles, we determined that *n*-alkanes were totally degraded after 453 days of incubation under mesophilic conditions (Fig. 2A). All of *n*-alkanes with chain length greater than 23 were totally degraded at 55°C after 303 days of incubation, and those less than 23 were incompletely degraded (Fig. 2B). The extent of degradation was also assessed by the pristane/heptadecane (Pr/*n*C_17_) ratio, which increased from 0.317±0.009 at day 155 to 1.557 at day 330 under mesophilic conditions, and finally approached infinity with the complete degradation of *n*-heptadecane after 450 days of incubation. The value of the Pr/*n*C_17_ ratio increased to 0.706±0.035 at day 303 in the thermophilic oil-degrading consortium. On the contrary, the ratio remained constant in the controls without microbial incubation at both temperatures (Table 1). In addition, the preferential degradation of longer-chain *n*-alkanes was observed at both temperatures (Fig. 2 and Table 1). GC analysis showed that the chromatograms of saturated hydrocarbons shifted from a unimodal type to bimodal patterns after 245 days of incubation at 35°C, the bimodal pattern was well-established after 330 days of incubation (Fig. 2A).

**Table 1:** Changes in group composition and biomarker ratios of crude oil during methanogenic degradation.

Incubation temperature	Time (d)	Group composition of crude oil (%)					Biomarker of crude oil
		Non-hydrocarbons	Saturated hydrocarbons	Aromatic hydrocarbons	Asphaltene	Recovery efficiency (%)	Pr/*n*C_17_
	0	14.9	33.4	22.5	29.6	97.4	0.3
35°C	155	15.4±3.1	24.9±0.5	30.7±1.0	17.0±1.5	88.0	0.317±0.009
	245	18.7±1.3	23.1±0.5	30.8±2.8	16.9±2.3	89.6	0.390±0.046
	330	36.2±0.6	10.1±1.0	24.8±2.1	16.0±1.3	87.2	1.557^a^
	450	31.8±4.3	8.6±0.9	21.5±2.5	30.7±0.9	92.7	^b^
	Sterilized-330	28.9±3.0	25.2±1.2	22.5±2.0	12.4±1.9	89.0	0.347±0.005
55°C	203	18.0±3.4	23.1±0.7	30.0±3.4	17.4±1.6	88.6	0.301±0.008
	303	32.3±5.1	10.8±0.7	26.8±2.0	18.8±3.7	88.7	0.777±0.130
	Sterilized-303	31.3±10.6	21.6±2.8	21.8±2.8	14.5±4.9	89.2	0.3448 + 0.0008

Pr/*n*C_17_: pristane/*n*-heptadecane; three replicates in each time points with exception of original day 0. a *n*-heptadecane was only detected in one of the three replicates; b: *n*-heptadecane was not detected above the detection limit; Sterilized-330: crude oil sampled from the sterilized control group after 330 days of incubation at 35°C, Sterilized-303: crude oil sampled from the sterilized control group after 303 days of incubation at 55°C.

### Microbial community structure and dynamics

#### Archaeal domain

**Figure 3:**
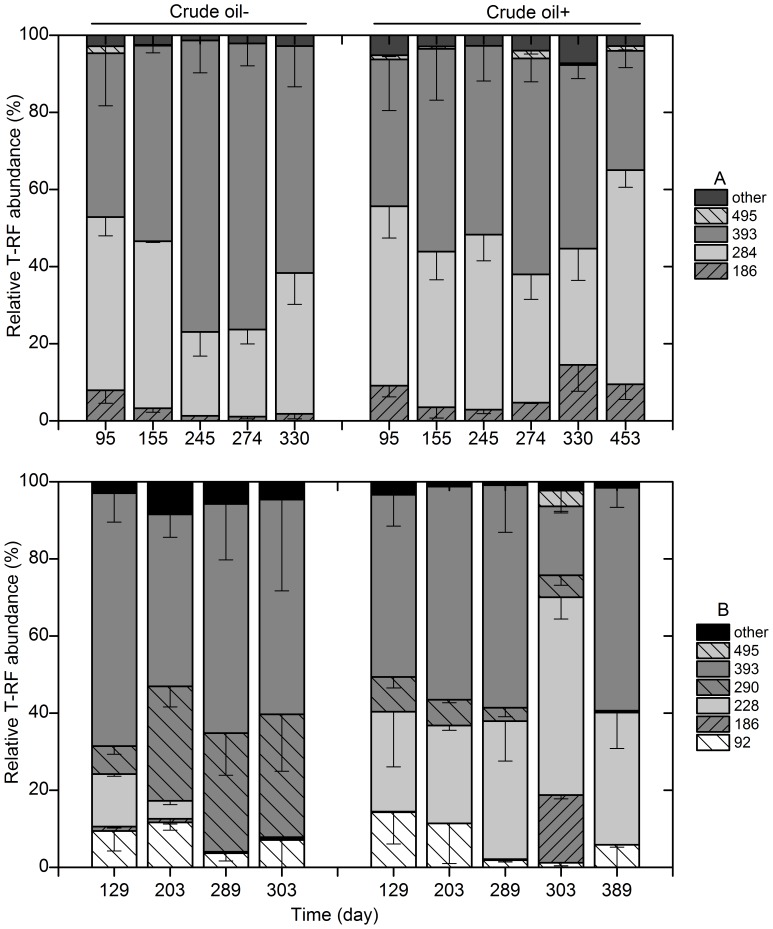
The archaeal T-RFLP profiles at 35°C (A) and 55°C (B). Crude oil -: consortium without crude oil addition; crude oil +: consortium with crude oil addition. Error bars represent the standard deviation, three replicates in crude oil-amended consortium and duplicates in crude oil-free consortium.

T-RFLP analysis of archaeal 16S rRNA genes revealed that the methanogenic crude oil-degrading cultures incubated at 35°C mainly consisted of T-RFs of 284-and 393-bp (78–94%). The community structure did not fluctuate much during 450 days of incubation, which was similar to the crude oil-free control (Fig. 3A). Under thermophilic conditions, the 228- and 393-bp T-RFs dominated in the crude oil-degrading cultures, which accounted for 47±16 and 35±21% of the population, respectively. During 389 days of incubation; the 290-bp T-RF became the second most dominant phylotype (24±19%) in the crude oil-free control after the most dominant T-RF of 393-bp (56±29%) (Fig. 3B).

**Figure 4:**
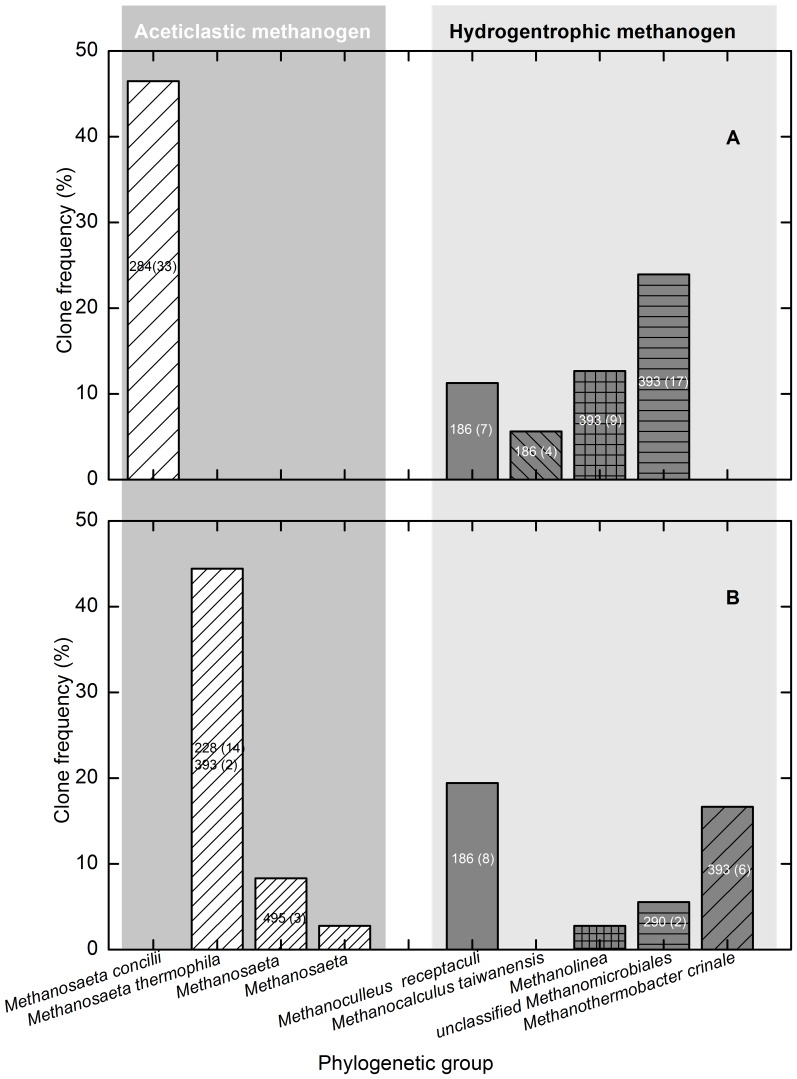
Phylogenetic affiliation of archaeal 16S rRNA gene sequences of the methanogenic crude oil-degrading consortia. A: 35°C; B: 55°C. The number in the column indicates the major OTUs represented by specific T-RFs, followed by the number in parentheses indicating the clone numbers of each OTU.

Two archaeal 16S rRNA gene clone libraries (L35A: total 71 clones retrieved from the mesophilic consortium; L55A: total 36 clones from the thermophilic consortium) were generated from the methanogenic crude oil-degrading consortia at 35 and 55°C, respectively, as shown in Table S1 in File S1. The analyses of rarefaction curves and Good coverage indexes revealed that saturation was reached (Fig. S2 and Table S1 in File S1). The predominant 284-bp T-RF retrieved from the mesophilic consortium was related to *Methanosaeta concilii* (>99% sequence similarity). The 186- and 393-bp T-RFs were related to the hydrogenotrophic *Methanomicrobiales* (Fig. 4A, Fig. S3 and Table S1 in File S1). Similarly, the 228- and 495-bp T-RFs in the thermophilic consortium were mainly related to *Methanosaeta thermophila* (91–99% sequence similarity). The 393- and 186-bp T-RFs were mainly related to H_2_-using *Methanothermobacter* and *Methanoculleus*, respectively (Fig. 4B, Fig. S3 and Table S1 in File S1).

#### Bacterial domain

**Figure 5:**
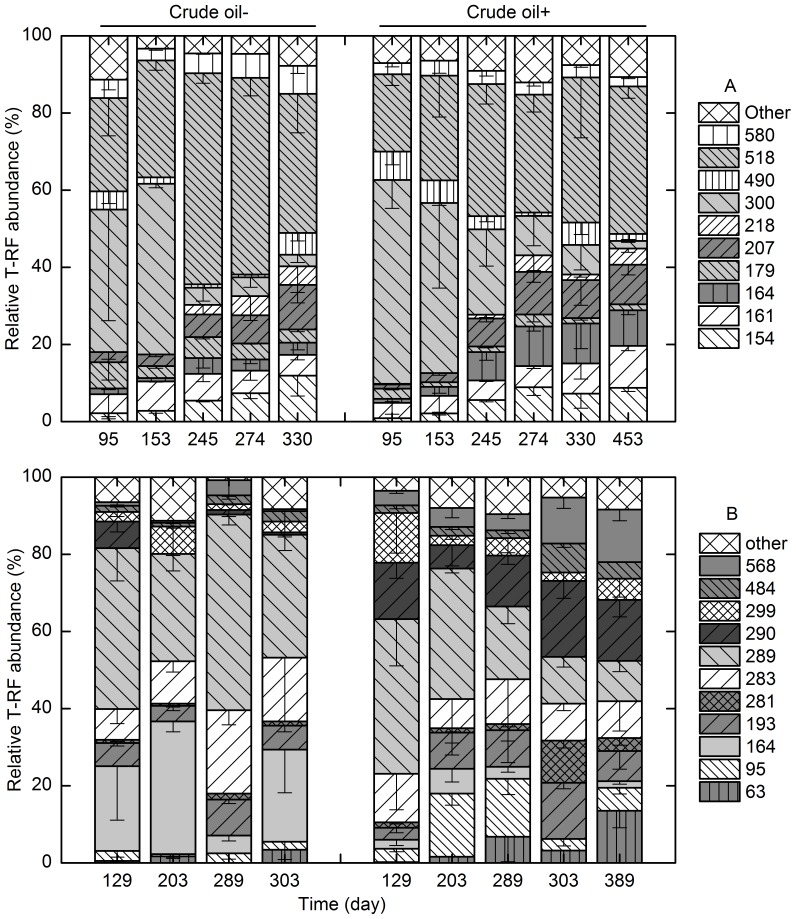
Bacterial T-RFLP profiles at 35°C (A) and 55°C (B). Crude oil -: consortium without crude oil addition; Crude oil +: crude oil amended consortium. Error bars represent the standard deviation, three replicates in crude oil-amended consortium and duplicates in crude oil-free consortium.

**Figure 6:**
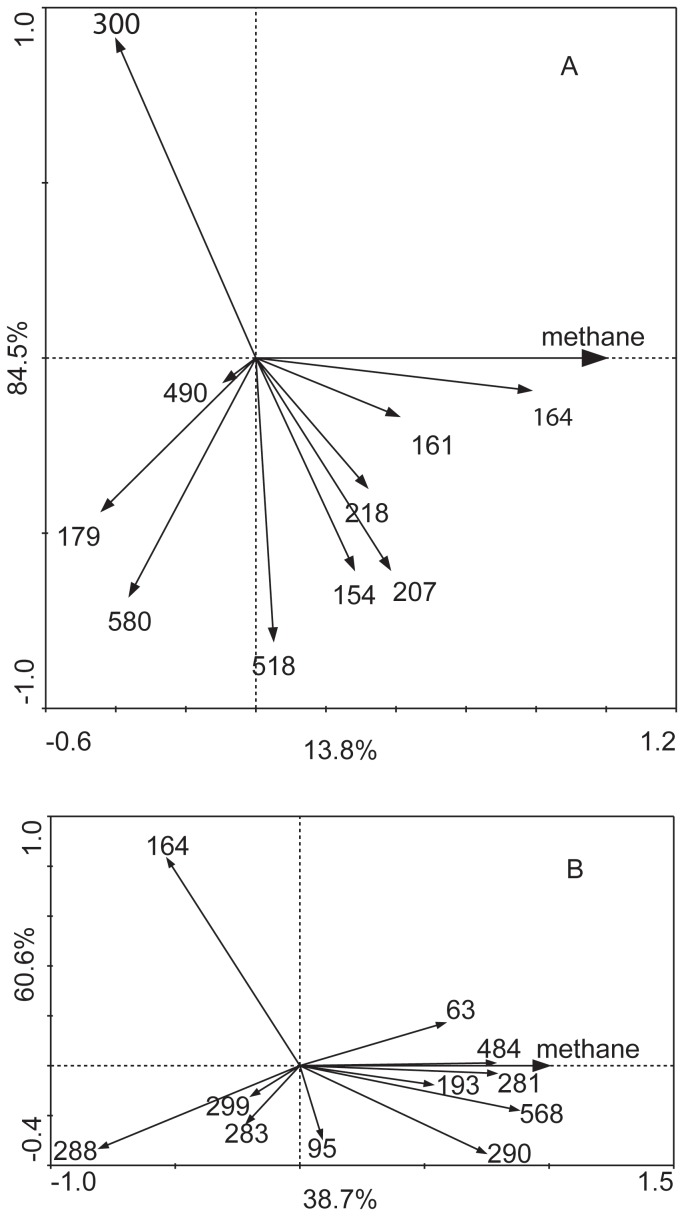
Redundancy analysis of bacterial TRFLP profiles versus methane production in methanogenic oil-degrading consortia at 35°C (A) and 55°C (B). Bold arrow denotes the explanatory variable of methane production. Values on the axes indicate the percentages of total variation explained by each axis.

The bacterial community structure was more diverse than the archaeal community structure, with 10–11 T-RFs routinely observed (Fig. 5). In the mesophilic cultures, The 161- and 164-bp T-RFs increased in relative abundance over time in the crude oil-amended cultures, compared with the crude oil-free control (Fig. 5A). Redundancy analysis revealed that the 154-, 161-, 164-, 207- and 218-bp T-RFs were positively associated with methane production in the mesophilic consortium (Fig. 6A). Redundancy analysis also revealed that the 63-, 193-, 281-, 290-, 484- and 568-bp T-RFs were highly correlated with methane production in the thermophilic consortium (Fig. 6B).

**Figure 7:**
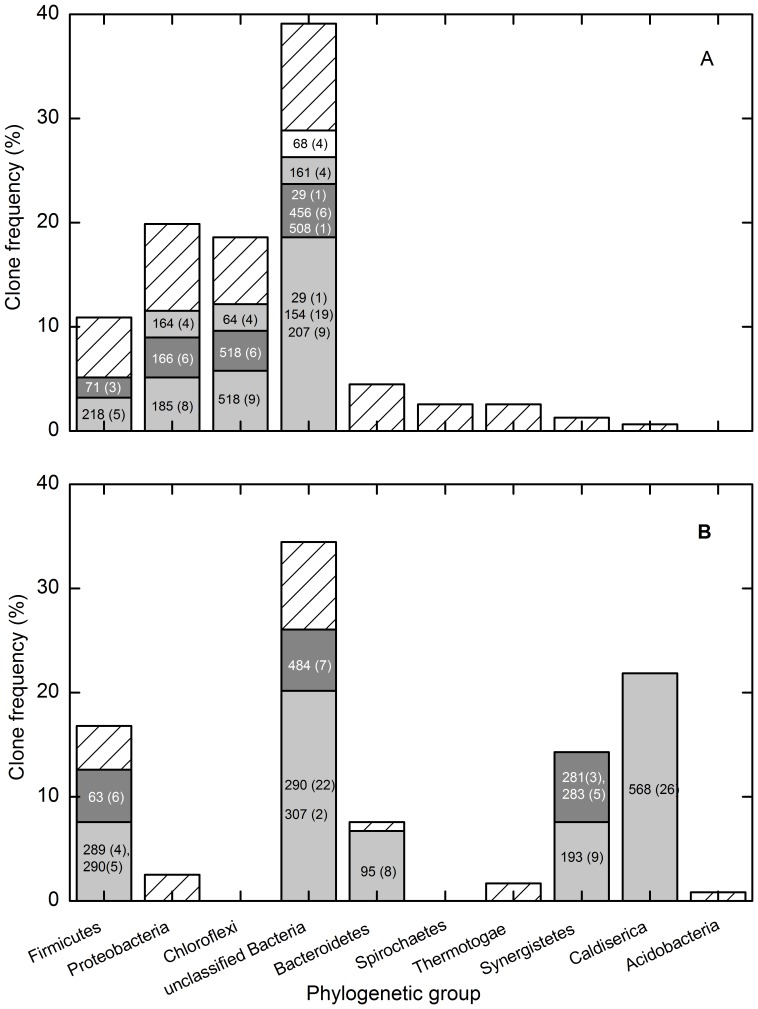
Phylogenetic affiliation of bacterial 16S rRNA gene sequences of the methanogenic crude oil-degrading consortia. A: 35°C; B: 55°C. The number in the column indicates the major OTUs represented by specific T-RFs, followed by the number in parentheses indicating the clone numbers of each OTU.

Two bacterial clone libraries (L35B: 156 clones from the mesophilic consortium; L55B: 119 clones from the thermophilic consortium) were also constructed from the same time points as for archaea. The rarefaction curves tended towards the saturation plateau (Fig. S2 in File S1), and coverage analysis suggested that 78.8 and 89.1% of predicted phylotypes were sampled from mesophilic and thermophilic consortia, respectively (Table S2 in File S1). The mesophilic bacterial community was mainly composed of unclassified *Bacteria* (39.1%), *Proteobacteria* (19.9%), *Chloroflexi* (18.6%) and *Firmicutes* (10.9%) (Fig. 7A, Fig. S4 and Table S2 in File S1). The bacterial community at 55°C was significantly different from that at 35°C, which mostly consisted of unclassified *Bacteria* (34.5%), *Caldisericales* (21.8%), *Firmicutes* (16.8%), *Synergistetes* (14.3%) and *Bacteroidetes* (7.5%) (Fig. 7B, Fig. S4 and Table S3 in File S1).

The combined analysis of T-RFLP profiles and clone library data revealed that the 161-bp T-RF presumably represented *in silico* T-RFs of 160, 161 and 162 bp as single clones, which could not be discriminated through capillary electrophoresis. The 160-bp T-RF represented uncultured bacteria with 86% sequence similarity to *Syntrophomonas zehnderi* [48], and the 162-bp T-RF represented uncultured members with 94–95% sequence similarity to syntrophic phenol degrading bacterium *Syntrophorhabdus aromaticivorans* (Fig. S4 and Table S2 in File S1) [49]. The 161-bp T-RF represented members of uncultured candidate division WWE1 bacteria, with 94 – 100% sequence similarity to Candidatus *Cloacamonas acidaminovorans* (Fig. S4 and Table S2 in File S1) [50]. The 154- and 207-bp T-RFs represented uncultured candidate division JS1 bacterium. The 164-bp T-RF was most closely related to uncultured candidate division JS1, *Syntrophaceae* or *Desulfovibrionaceae* (Fig. S4 and Table S2 in File S1). In addition, the dominant 518-bp T-RF mainly represented uncultured members of *Chloroflexi* (Fig. S4 and Table S2 in File S1). The 300-bp T-RF representing *Soehngenia saccharolytica*-related microorganisms dominated at the early stage of incubation but became less abundant during the last 200 days of incubation (Fig. 5A and Table S2 in File S1). The 185-bp T-RF, the third most dominant OTU, was distantly related to *Smithella propionica* (92% sequence similarity) [51], and the 166-bp T-RF, the fifth most dominant OTU, shared 94% sequence similarity with *Desulfatibacillum alkenivorans* (Fig. S4 and Table S2 in File S1) [52], but these two fragments were not detected in the T-RFLP profile at high abundance (>2%).

In the thermophilic cultures, the 63-bp T-RF represented members of *Firmicutes*, which exhibited 91% sequence similarity to the thermophilic acetogen *Moorella thermoacetica* (Fig. S4 and Table S3 in File S1) [53]. The 193- and 281-bp T-RFs represented members of *Synergistetes* (two taxa), which shared 88 - 98% sequence similarity with *Anaerobaculum thermoterrenum* [54]. The second most dominant OTU (22 clones) represented by the 290-bp T-RF, shared 81% sequence similarity with *Thermotoga maritima* [55], which could be clustered into “Shengli cluster” [28]. The other OTU (5 clones) represented by the 290-bp T-RF was closely related to protein-degrading bacterium *Coprothermobacter proteolyticus* (99% sequence similarity) [56],[57]. The 484-bp T-RF represented unclassified bacteria, which shared 83.8% sequence similarity with *Carboxydothermus hydrogenoformans* (Fig. S4 and Table S3 in File S1) [58]. The 568-bp T-RF represented the dominant OTU (84% sequence similarity to *Caldisericum exile*) (Fig. S4 and Table S3 in File S1) [59].

## Discussion

Research studies on the microbial oxidation of crude oil coupled to methane production have been well documented. The methane yield varied from 0.6 to 10.0 mmol per gram of oil from various oilfields [6],[9],[11]–[13], However, research on the effect of temperature on the methane potential and kinetics of crude oil degradation is scarce. In this study, oily sludge microbes collected from the Shengli oilfield possessed the ability to degrade crude oil *n*-alkanes and generate methane at both mesophilic (35°C) and thermophilic (55°C) temperatures. The mesophilic consortium, amended with the same amount of crude oil, could produce more methane than that at 55°C, but with a slower methane production rate. These results indicate that the oily sludge microcosms could degrade crude oil via methanogenesis over a wide temperature range. The difference in methane production between the mesophilic and thermophilic cultures may be attributed to the degree of crude oil degradation, and is probably correlated with the loss of *n*-alkanes. All *n*-alkanes completely disappeared at 35°C, while *n*-alkanes with chain lengths from C_11_ to C_23_ were incompletely degraded at 55°C. Crude oil is a complex mixture consisting of various *n*-alkanes, of which shorter chain *n*-alkanes are generally degraded faster than longer ones in previous reports [2],[5]. Siddique et al. [15],[16] reported that methanogenic microcosms enriched from the same mature fine tailings possessed different degradation patterns for short and long chain *n*-alkanes. The preferential degradation of mid- and high-range alkanes has also been reported [17]–[19], which differed from the expected pattern under aerobic conditions. Surprisingly, a relative enrichment of shorter *n*-alkanes was also detected [18],[19], which was questioned by Galperin *et al*. [60] because of the unusual progression of *n*-alkane degradation. In this study, the preferential degradation of longer-chain *n*-alkanes was also observed at both temperatures tested, which is similar to results obtained in previous studies [16]–[19]. This result confirms the general progression of crude oil degradation under methanogenic conditions, however, a relative accumulation of shorter-chain *n*-alkanes was not observed.

The methanogenic degradation of hydrocarbons requires syntrophic cooperation of bacterial and archaeal communities, and thermodynamics analysis revealed five possible pathways for the conversion of hydrocarbons into methane [24]. The concurrence of acetoclastic and hydrogenotrophic methanogens, but with different methanogenic community structures, was observed at both temperatures in this study, which is similar to our previously reported consortia using hexadecane as substrate [38]. The dominant archaeal community during consecutive transfer and incubation with hexadecane shifted from acetoclastic *Methanosaeta* to hydrogenotrophic *Methanoculleus* [38]. *Methanoculleus* spp. were further reported as the dominant methane producers through DNA-SIP [27]. Methane production from crude oil was proposed to be generated through syntrophic acetate oxidation coupled to hydrogenotrophic *Methanothermobacter* under thermophilic conditions [11]. Similarly, the hydrogenotrophic *Methanothermobacter* has also been revealed as the dominant archaeal population in the hexadecane degrading consortium [28],[33]. *Methanosaeta* spp., represented by the 228 bp T-RF, dominated in the thermophilic crude oil-degrading consortium, which suggests their potential role in methane production through acetate fermentation. Interestingly, it has been reported that archaeal populations in crude oil enrichment cultures were mainly composed of non-methanogenic archaea [29],[61]. These results suggest that further research is needed for characterization of the relative contribution of archaea to methane production during crude oil degradation.

The complex components of crude oil provide a vast range of substrates for development of a complex microbial community. Knowledge about the key players initiating methanogenic hydrocarbon degradation is limited, as bacteria that degrade and grow on hydrocarbons via methanogenesis have to deal with the unfavorable energetics of the conversion processes [8],[24],[62]. The uncultured JS1 lineage and members of the Chloroflexi have been identified as the major clades of bacteria in the mesophilic consortium, which have been widely detected in oil-impacted environments and proposed to play a role in hydrocarbon degradation [63],[64]. Direct evidence for methanogenic degradation of *n*-alkanes by these two phylotypes has not been reported. The third major bacterial phylotype was related to *Proteobacteria* and was mainly composed of members of *Desulfobacteraceae*, *Syntrophaceae* and *Syntrophorhabdus*. The *Desulfobacteraceae*-affiliated member was most closely related to *D. alkenivorans*, which could oxidize alkanes with sulfate as an electron acceptor, or coupled to methane production [25],[65]. The dominant *Syntrophaceae*-related OTU (type clone L35B_12) in this consortium shared 92% sequence similarity to syntrophic propionate oxidation bacterium *S. propionica* [51],[66], and may represent an uncultured alkane degrader species. The *Syntrophaceae*-affiliated members have been identified as key players associated with alkane degradation under methanogenic conditions using culture-independent methods [26],[27]. The *ass*A related gene observed in the other *Syntrophaceae*-affiliated members may indicate fumarate addition to alkanes [67],[68]. Members of this family have also been detected in a number of methanogenic alkane-degrading cultures [7],[12],[13],[15],[26],[27],[31],[69],[70], suggesting their ecophysiological role in the anaerobic alkane degradation process. *S. aromaticivorans* species could use phenol, p-cresol, isophthalate, benzoate, and 4-hydroxybenzoate in syntrophic association with a hydrogenotrophic methanogen with an optimum growth temperature range of 35–37°C [49], which indicates that the *Syntrophorhabdus*-related members may use aromatic hydrocarbons in crude oil to grow, and enhance methane production under mesophilic conditions. Further characterization of aromatic degradation by this consortium is beyond the scope of the study.

The thermophilic bacterial community structure is different from that at 35°C, which was mainly grouped into *Caldisericales* and “Shengli cluster” [28]. The *Caldisericales*-related members (type clone L55B_6) shared only 84% sequence similarity with *C. exile*, which grows anaerobically with yeast extract and sulfur compounds with thiosulfate, sulfite and elemental sulfur as electron acceptors [59]. The relatively few environmental clones related to *C. exile* were detected in hot spring sediment (GenBank *No*.: FJ638586), an anaerobic bioreactor (GenBank *No*.: EF515667) and terephthalate-degrading sludge [71]. The other dominant phylotype (type clone: L55B_1) exhibited 81% sequence similarity with extreme thermophile *T. maritima* [55], and shared 95% sequence similarity with representative clone BSK_100 of “Shengli Cluster” retrieved from a thermophilic hexadecane-degrading consortium, which was proposed to be involved in thermophilic hexadecane degradation [28]. The role of other observed less dominant bacterial phylotypes in both consortia is not clear. However, their persistent presence suggests that they are probably associated with the process of hydrocarbon degradation, possibly via multiple syntrophic interactions [72].

In summary, this present study shows that oily sludge microbes from Shengli oilfield possess the ability to degrade crude oil *n*-alkanes coupled to methane production at 35 and 55°C. The preferential degradation of long chain alkanes by oily sludge microbes was observed at both temperatures. Both acetoclastic and hydrogenotrophic methanogens are likely to play a role in methane production during oil degradation under mesophilic and thermophilic conditions. The dominant bacterial community structure is different between the two consortia, suggesting the adaptation of oily sludge microbes to the shift in temperature by selective growth of specific groups of microbes. [62] [14] [60].

## Supporting Information

File S1 **Figure S1,** Time course of methane production of the pre-enrichment cultures incubated at 35°C (A) and 55°C (B). The square and rhombus represent the pre-enichment cultures incubated at 35°C, The triangles represent the pre-enrichment cultures incubated at 55°C. The arrows indicates the sampling points for transfer incubation of the pre-enrichment cultures. **Figure S2,** Rarefaction curves constructed from archaeal and bacterial 16S rRNA gene libraries based on OTU cutoff of equal or higher 97%. **Figure S3,** Phylogenetic tree based on archaeal 16S rRNA gene sequences from representative clones of each OTUs, related type strains and environmental clones using neighbor-joining analysis of 779-nt alignment. Representative clones from the mesophilic consortium are indicated in red, followed by *in silico* T-RFs and clone numbers, and the representative clones from the thermophilic consortium are indicated in blue, followed by *in silico* T-RFs and clone numbers. **Figure S4,** Phylogenetic tree based on bacterial 16S rRNA gene sequences from representative clones of each OTUs and related strains and environmental clones using neighbor-joining analysis of 698-nt alignment. Representative clones from the mesophilic consortium are indicated in red, followed by *in silico* T-RFs and clone numbers, and the representative clones from the thermophilic consortium are indicated in blue, followed by *in silico* T-RFs and clone numbers, the OTUs less than 3 clones in the mesophilic consortium and 2 in the thermophilic consortium were not shown in the phylogenetic tree. *Methanothermobacter crinale* (EF554596) was used as outgroup. The scale bar represents 2% sequence divergence. **Table S1,** Phylogenetic affiliation of archaeal 16S rRNA genes and corresponding theoretical T-RFs retrieved from methanogenic oil-degrading consortia at 35 and 55°C, respectively. **Table S2,** Phylogenetic affiliation of bacterial 16S rRNA genes and corresponding theoretical T-RFs retrieved from mesophilic methanogenic oil-degrading consortium. **Table S3,** Phylogenetic affiliation of bacterial 16S rRNA gene and corresponding theoretical T-RFs retrieved from thermophilic methanogenic oil-degrading consortium.
(DOCX)
