# Supplementary material for: Progressive Degradation of Crude Oil n-Alkanes Coupled to Methane Production under Mesophilic and Thermophilic Conditions
Source: PLoS One. 2014 Nov 19;9(11):e113253. doi: 10.1371/journal.pone.0113253 (PMC4237390; doi:10.1371/journal.pone.0113253)
Supplement: File S1 — Figure S1, Time course of methane production of the pre-enrichment cultures incubated at 35°C (A) and 55°C (B). The square and rhombus represent the pre-enichment cultures incubated at 35°C, The triangles represent the pre-enrichment cultures incubated at 55°C. The arrows indicates the sampling points for transfer incubation of the pre-enrichment cultures. Figure S2, Rarefaction curves constructed from archaeal and bacterial 16S rRNA gene libraries based on OTU cutoff of equal or higher 97%. Figure S3, Phylogenetic tree based on archaeal 16S rRNA gene sequences from representative clones of each OTUs, related type strains and environmental clones using neighbor-joining analysis of 779-nt alignment. Representative clones from the mesophilic consortium are indicated in red, followed by in silico T-RFs and clone numbers, and the representative clones from the thermophilic consortium are indicated in blue, followed by in silico T-RFs and clone numbers. Figure S4, Phylogenetic tree based on bacterial 16S rRNA gene sequences from representative clones of each OTUs and related strains and environmental clones using neighbor-joining analysis of 698-nt alignment. Representative clones from the mesophilic consortium are indicated in red, followed by in silico T-RFs and clone numbers, and the representative clones from the thermophilic consortium are indicated in blue, followed by in silico T-RFs and clone numbers, the OTUs less than 3 clones in the mesophilic consortium and 2 in the thermophilic consortium were not shown in the phylogenetic tree. Methanothermobacter crinale (EF554596) was used as outgroup. The scale bar represents 2% sequence divergence. Table S1, Phylogenetic affiliation of archaeal 16S rRNA genes and corresponding theoretical T-RFs retrieved from methanogenic oil-degrading consortia at 35 and 55°C, respectively. Table S2, Phylogenetic affiliation of bacterial 16S rRNA genes and corresponding theoretical T-RFs retrieved from mesophilic methanogenic oil-degra [file pone.0113253.s001.docx]

### Supporting Information Legends

**Fig. S1.** Time course of methane production of the pre-enrichment cultures incubated at 35°C (A) and 55°C (B). The square and rhombus represent the pre-enichment cultures incubated at 35°C, The triangles represent the pre-enrichment cultures incubated at 55°C. The arrows indicates the sampling points for transfer incubation of the pre-enrichment cultures.

**Fig. S2.** Rarefaction curves constructed from archaeal and bacterial 16S rRNA gene libraries based on OTU cutoff of equal or higher 97%.

**Fig. S3.** Phylogenetic tree based on archaeal 16S rRNA gene sequences from representative clones of each OTUs, related type strains and environmental clones using neighbor-joining analysis of 779-nt alignment. Representative clones from the mesophilic consortium are indicated in red, followed by *in silico* T-RFs and clone numbers, and the representative clones from the thermophilic consortium are indicated in blue, followed by *in silico* T-RFs and clone numbers.

**Fig. S4.** Phylogenetic tree based on bacterial 16S rRNA gene sequences from representative clones of each OTUs and related strains and environmental clones using neighbor-joining analysis of 698-nt alignment. Representative clones from the mesophilic consortium are indicated in red, followed by *in silico* T-RFs and clone numbers, and the representative clones from the thermophilic consortium are indicated in blue, followed by *in silico* T-RFs and clone numbers, the OTUs less than 3 clones in the mesophilic consortium and 2 in the thermophilic consortium were not shown in the phylogenetic tree.*Methanothermobacter crinale* (EF554596) was used as outgroup. The scale bar represents 2% sequence divergence.

**Table S1.** Phylogenetic affiliation of archaeal 16S rRNA genes and corresponding theoretical T-RFs retrieved from methanogenic oil-degrading consortia at 35 and 55°C, respectively.

**Table S2**. Phylogenetic affiliation of bacterial 16S rRNA genes and corresponding theoretical T-RFs retrieved from mesophilic methanogenic oil-degrading consortium.

**Table S3.** Phylogenetic affiliation of bacterial 16S rRNA gene and corresponding theoretical T-RFs retrieved from thermophilic methanogenic oil-degrading consortium.

**Fig. S1**


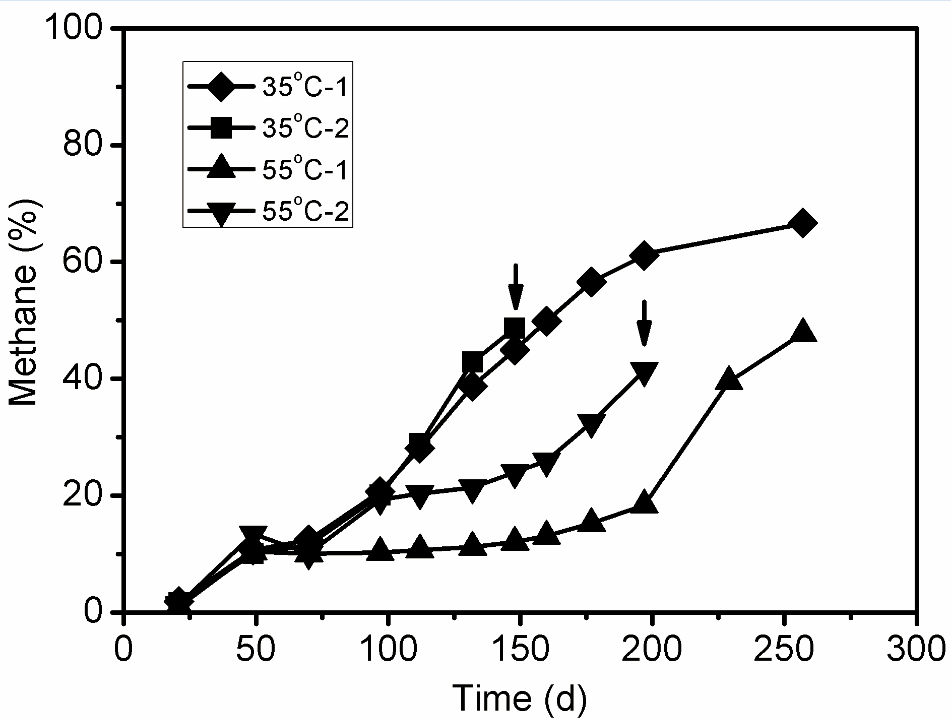


**Fig. S2**


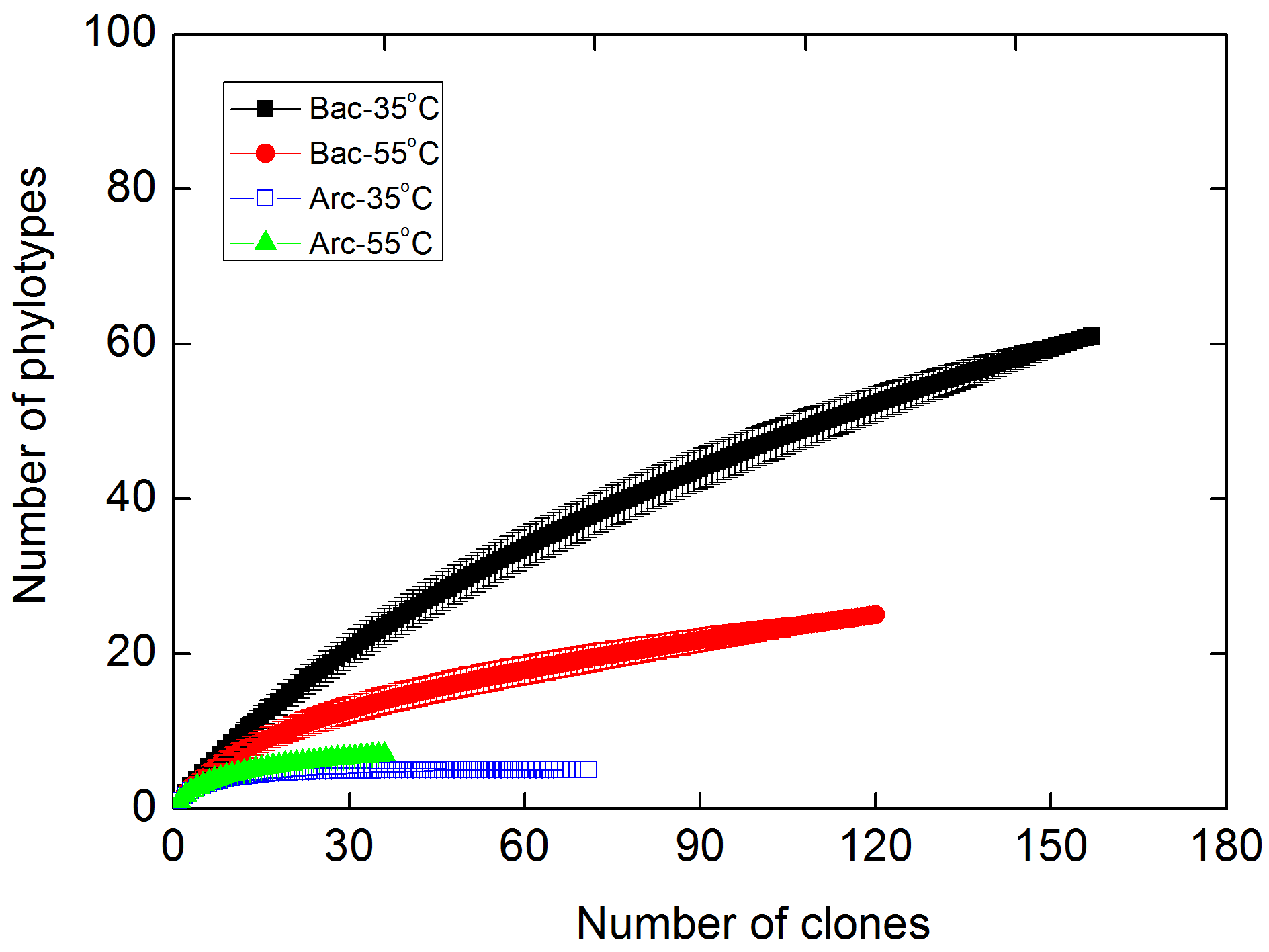


**Fig. S3**


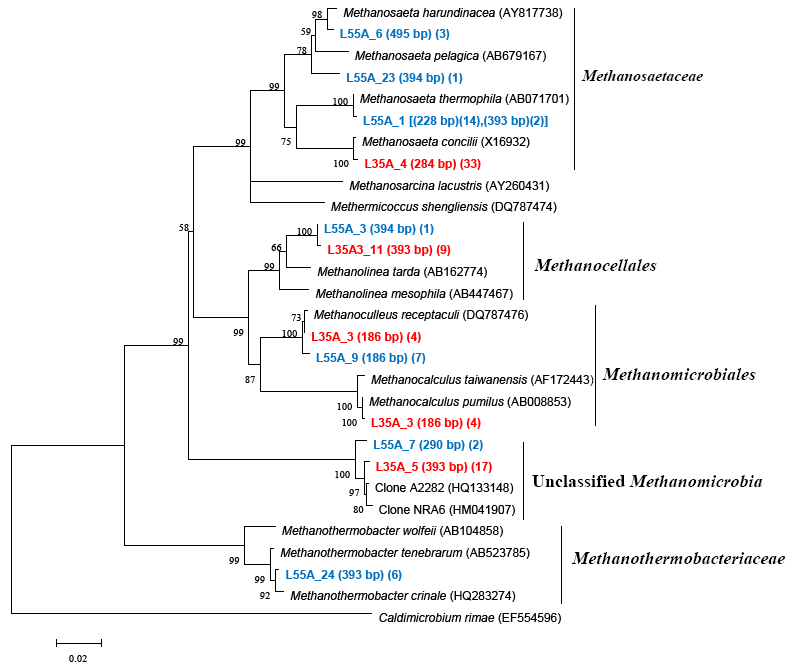


**Fig. S4**


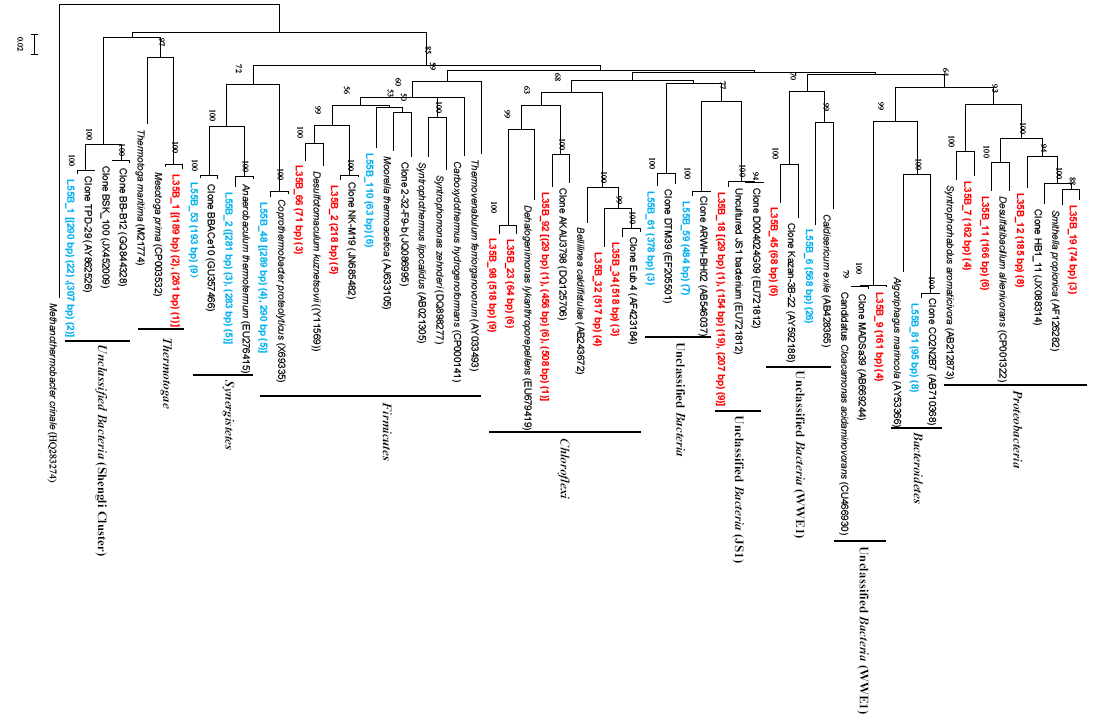


**Table S1**.

| **Phylogenetic group** | **L35A** | | |  | **L55A** | |  | **Type clone** | **The most similar sequence according to RDP 10** | |
| --- | --- | --- | --- | --- | --- | --- | --- | --- | --- | --- |
|  |  | ***In silico* T-RF (bp) ^#^** | ***No*. of clones**  **per OTU^#^** |  | ***In silico* T-RF (bp)^#^** | ***No*. of clones per OTU^#^** |  |  | **Closest relative species** | **Similarity** |
| *Methanosaetaceae* |  | 284 | 33 |  |  | 0 |  | L35A_4 | *Methanosaeta concilii*; X51423 | 0.996 |
|  |  |  | 0 |  | 228 (14),393 (2)^&^ | 16 |  | L55A_1 | *Methanosaeta thermophile*; AB071701 | 0.997 |
|  |  |  | 0 |  | 495 | 3 |  | L55A_6 | *Methanosaeta thermophile*; AB071701 | 0.913 |
|  |  |  | 0 |  | 495 | 1 |  | L55A_23 | *Methanosaeta harundinacea*; AY817738 | 0.967 |
| *Methanomicrobia* |  | 186 | 8 |  | 186 | 7 |  | L35A_8 | *Methanoculleus receptaculi*; DQ787476 | 0.995 |
|  |  | 186 | 4 |  |  | 0 |  | L35A_3 | *Methanocalculus taiwanensis*; AF411470 | 0.992 |
|  |  | 393 | 9 |  | 394 | 1 |  | L35A3_11 | *Methanolinea tarda*; AB162774 | 0.958 |
|  |  | 393 | 17 |  | 290 | 2 |  | L35A_5 | *Methanolinea tarda*; AB162774 | 0.817 |
| *Methanobacteriaceae* |  |  | 0 |  | 393 | 6 |  | L55A_24 | *Methanothermobacter crinale*; HQ283273 | 0.990 |
| Total clones |  |  | 71 |  |  | 36 |  |  |  |  |
| good coverage |  |  | 1 |  |  | 0.946 |  |  |  |  |
| Chao I |  |  | 3 (3 ± 3.1) |  |  | 7.5 (7.0 ± 15.3) |  |  |  |  |

&: number in the parentheses indicates numbers of clones. #: OTU: >97% sequences similarity.

**Table S2**

| **Phylogenetic group**  **(*No*. of clones)** | ***In silico* T-RFs** | ***No*. of Clones per OTU^#^** | **Type clone** | **The most similar sequence according to RDP 10** | |
| --- | --- | --- | --- | --- | --- |
|  |  |  |  | **Closest relative species** | **Similarity** |
| *Chloroflexi* (29) | 173 | 1 | L35B_110 | *Bellilinea caldifistulae*; AB243672 | 0.969 |
|  | **517** | **4** | **L35B_32** | ***Bellilinea caldifistulae*; AB243672** | **0.897** |
|  | **518** | **3** | **L35B_34** | ***Bellilinea caldifistulae*; AB243672** | **0.917** |
|  | 522 | 1 | L35B_120 | *Bellilinea caldifistulae*; AB243672 | 0.885 |
|  | 580 | 1 | L35B_57 | *Bellilinea caldifistulae*; AB243672 | 0.896 |
|  | 372 | 2 | L35B_54 | *Bellilinea caldifistulae*; AB243672 | 0.926 |
|  | **64** | **6** | **L35B_23** | ***Dehalogenimonas lykanthroporepellens*; EU679419** | **0.843** |
|  | 518 | 1 | L35B_78 | *Dehalogenimonas lykanthroporepellens*; EU679419 | 0.846 |
|  | **518** | **9** | **L35B_98** | ***Dehalogenimonas lykanthroporepellens*; EU679419** | **0.85** |
|  | 525 | 1 | L35B_126 | *Dehalogenimonas lykanthroporepellens*; EU679419 | 0.834 |
| *Proteobacteria* (31) | **166** | **6** | **L35B_11** | ***Desulfatibacillum alkenivorans*; AY493562** | **0.944** |
|  | 166 | 2 | L35B_29 | *Desulfatibacillum alkenivorans*; AY493562 | 0.95 |
|  | 164 | 1 | L35B_20 | *Desulfovibrio marrakechensis*; AM947130 | 0.928 |
|  | 131 | 2 | L35B_22 | *Pelobacter venetianus*; U41562 | 0.97 |
|  | **74** | **3** | **L35B_19** | ***Smithella propionica*; AF126282** | **0.97** |
|  | 164 | 1 | L35B_15 | *Smithella propionica*; AF126282 | 0.92 |
|  | **185** | **8** | **L35B_12** | ***Smithella propionica*; AF126282** | **0.924** |
|  | 184 | 1 | L35B_121 | *Smithella propionica*; AF126282 | 0.917 |
|  | 131 | 2 | L35B_31 | *Syntrophorhabdus aromaticivorans*; AB212873 | 0.938 |
|  | 162 | 1 | L35B_127 | *Syntrophorhabdus aromaticivorans*; AB212873 | 0.951 |
|  | **162** | **4** | **L35B_7** | ***Syntrophorhabdus aromaticivorans*; AB212873** | **0.942** |
| *Thermotogae* (4) | 265 | 1 | L35B_8 | *Petrotoga olearia*; AJ311703 | 0.994 |
|  | **189 (2),**  **261 (1)** | **3** | **L35B_1** | ***Mesotoga prima;* CP003532** | **0.993** |
| *Bacteroidetes* (7) | 91 | 1 | L35B_21 | *Pontibacter actiniarum*; AY989908 | 0.861 |
|  | 93 | 2 | L35B_13 | *Olivibacter sitiensis*; DQ421387 | 0.849 |
|  | 93 | 1 | L35B_72 | *Prolixibacter bellariivorans*; AY918928 | 0.892 |
|  | 95 | 2 | L35B_95 | *Algoriphagus aquatilis*; EU313811 | 0.804 |
|  | 538 | 1 | L35B_144 | *Prolixibacter bellariivorans*; AY918928 | 0.89 |
| *Firmicutes* (17) | **71** | **3** | **L35B_66** | ***Desulfotomaculum kuznetsovii*; Y11569** | **0.952** |
|  | 152 | 1 | L35B_80 | *Pelotomaculum propionicicum*; AB154390 | 0.971 |
|  | **218** | **5** | **L35B_2** | ***Desulfotomaculum thermobenzoicum*; AJ294429** | **0.906** |
|  | 170 | 1 | L35B_140 | *Soehngenia saccharolytica*; AY353956 | 0.961 |
|  | 300 | 2 | L35B_25 | *Soehngenia saccharolytica*; AY353956 | 0.994 |
|  | 63 | 2 | L35B_146 | *Moorella thermoacetica*; AY656675 | 0.904 |
|  | 146 | 1 | L35B_10 | *Moorella glycerini*; U82327 | 0.913 |
|  | 312 | 1 | L35B_30 | *Moorella glycerini*; U82327 | 0.88 |
|  | 289 | 1 | L35B_119 | *Coprothermobacter proteolyticus*; X69335 | 0.978 |
| *Spirochaetes* (4) | 161 | 1 | L35B_108 | *Spirochaeta smaragdinae*; U80597 | 0.891 |
|  | 212 | 2 | L35B_104 | *Treponema primitia*; AF09325 | 0.880 |
|  | 282 | 1 | L35B_101 | *Spirochaeta coccoides*; AJ698092 | 0.877 |
| *Synergistetes* (2) | 177 | 1 | L35B_49 | *Aminobacterium colombiense*; AF069287 | 0.903 |
|  | 290 | 1 | L35B_125 | *Anaerobaculum mobile*; AJ243189 | 0.886 |
| *Caldiserica* (1) | 570 | 1 | L35B_61 | *Caldisericum exile*; AB428365 | 0.82 |
| Uncultured division JS1 (34) | **29 (1),**  **154 (19),**  **207 (9)** | **29** | **L35B_18** | ***Syntrophomonas zehnderi*; DQ898277** | **0.854** |
|  | 207 | 1 | L35B_117 | *Syntrophomonas zehnderi*; DQ898277 | 0.855 |
|  | 339 | 1 | L35B_43 | *Syntrophomonas zehnderi*; DQ898277 | 0.855 |
|  | 160 | 1 | L35B_94 | *Syntrophomonas zehnderi*; DQ898277 | 0.855 |
|  | 164 | 2 | L35B_36 | *Syntrophomonas zehnderi*; DQ898277 | 0.856 |
| WWE1 bacterium (5) | 161 | 1 | L35B_84 | Candidatus Cloacamonas acidaminovorans; CU466930 | 0.946 |
|  | **161** | **4** | **L35B_9** | **Candidatus Cloacamonas acidaminovorans; CU466930** | **0.993** |
| unclassified Bacteria (22) | 65 | 1 | L35B_137 | *Dehalogenimonas lykanthroporepellens*; EU679419 | 0.861 |
|  | **68** | **4** | **L35B_45** | ***Moorella glycerini*; U82327** | **0.819** |
|  | 83 | 1 | L35B_88 | *Thermaerobacter subterraneus*; AF343566 | 0.803 |
|  | 125 | 2 | L35B_67 | *Anaerovibrio lipolyticus*; AB034191 | 0.83 |
|  | 195 | 1 | L35B_147 | *Verrucomicrobium spinosum*; X90515 | 0.814 |
|  | 230 | 1 | L35B_26 | *Halobacillus litoralis*; X94558 | 0.796 |
|  | 298 | 1 | L35B_76 | *Caloramator indicus*; X75788 | 0.853 |
|  | 547 | 1 | L35B_130 | *Clostridium acetobutylicum*; AE001437 | 0.756 |
|  | **29 (1),**  **456 (6),**  **508 (1)** | **8** | **L35B_92** | ***Syntrophothermus lipocalidus*; AB021305** | **0.836** |
|  | 378(1),  379(1) | 2 | L35B_71 | *Thermovenabulum ferriorganovorum*; AY033493 | 0.838 |
| Total clones |  | 156 |  |  |  |
| good coverage |  | 0.788 |  |  |  |
| Chao I |  | 104.2 (79.2 ± 163.6) |  |  |  |

**^#^:** OTU: >97% sequences similarity.

**Table S3**

| **Phylogenetic group** | ***In silico* TRFs** | ***No*. of clones per OTU^#^** | **Type clone** | **The most similar sequence according to RDP 10** | |
| --- | --- | --- | --- | --- | --- |
|  |  |  |  | **Closest relative species** | Similarity |
| *Acidobacteria* (1) | 266 | 1 | L55B_105 | *Thermaerobacter nagasakiensis*; AB061441 | 0.857 |
| *Bacteroidetes* (9) | 202 | 1 | L55B_108 | *Chryseobacterium pallidum*; AM232809 | 0.9 |
|  | **95** | **8** | **L55B_81** | ***Algoriphagus marincola*; AY533663** | **0.807** |
| *Proteobacteria* (3) | 299 | 1 | L55B_123 | *Enhydrobacter aerosaccus*; AJ550856 | 0.994 |
|  | 491 | 1 | L55B_16 | *Acinetobacter schindleri*; AJ278311 | 0.993 |
|  | 401 | 1 | L55B_50 | *Brucella pinnipedialis*; AM158981 | 0.993 |
| *Synergistetes* (17) | **281 (3),**  **283 (5)** | **8** | **L55B_2** | ***Anaerobaculum thermoterrenum*; U50711** | **0.986** |
|  | **193** | **9** | **L55B_53** | ***Anaerobaculum thermoterrenum*; U50711** | **0.885** |
| *Caldiserica* (26) | **568** | **26** | **L55B_6** | ***Caldisericum exile*; AB428365** | **0.835** |
| *Thermotogae* (2) | 140 | 1 | L55B_22 | *Thermotoga subterranea*; U22664 | 0.993 |
|  | 133 | 1 | L55B_72 | *Thermotoga hypogea*; U89768 | 0.989 |
| *Firmicutes* (20) | **63** | **6** | **L55B_110** | ***Moorella thermoacetica*; AY656675** | **0.911** |
|  | 209 | 1 | L55B_127 | *Thermoanaerobacter acetoethylicus* | 0.912 |
|  | 64 | 1 | L55B_38 | *Ammonifex thiophilus*; EF554597 | 0.911 |
|  | 150 | 1 | L55B_41 | *Moorella glycerini*; U82327 | 0.902 |
|  | 63 | 1 | L55B_44 | *Moorella thermoacetica*; AY656675 | 0.9 |
|  | 64 | 1 | L55B_101 | *Pelotomaculum thermopropionicum*; AB035723 | 0.988 |
|  | **289 (4),**  **290 (5)** | **9** | **L55B_48** | ***Coprothermobacter proteolyticus*; X69335** | **0.986** |
| unclassified *Bacteria* (41) | **290 (22),**  **307 (2)** | **24** | **L55B_1** | ***Thermotoga maritima*; M21774** | **0.806** |
|  | **484** | **7** | **L55B_59** | ***Carboxydothermus hydrogenoformans*; CP000141** | **0.838** |
|  | 484 | 2 | L55B_103 | *Ammonifex thiophilus*; EF554597 | 0.849 |
|  | **378** | **3** | **L55B_61** | ***Thermovenabulum ferriorganovorum*; AY033493** | **0.840** |
|  | 378 | 2 | L55B_114 | *Thermovenabulum ferriorganovorum*; AY033493 | 0.842 |
|  | 143 | 2 | L55B_62 | *Thermosulfidibacter takaii*; AB282756 | 0.818 |
|  | 142 | 1 | L55B_117 | *Thermoleophilum album*; AJ458462 | 0.875 |
| Total clones |  | 119 |  |  |  |
| good coverage |  | 0.891 |  |  |  |
| Chao I |  | 38.2 (28.4 ± 75.6) |  |  |  |

**^#^**: OTU: >97% sequences similarity.
